# Insights of Chinese herbal medicine for mitochondrial dysfunction in chronic cerebral hypoperfusion induced cognitive impairment: Existed evidences and potential directions

**DOI:** 10.3389/fphar.2023.1138566

**Published:** 2023-02-10

**Authors:** Yefei Wang, Ying Wang, Shixin Li, Huihui Jin, Jiayu Duan, Xiyue Lu, Yinglin Qin, Jiale Song, Xiaoshan Li, Xianglan Jin

**Affiliations:** ^1^ Graduate School, Beijing University of Chinese Medicine, Beijing, China; ^2^ Department of Neurology, Dongfang Hospital, Beijing University of Chinese Medicine, Beijing, China

**Keywords:** chronic cerebral hypoperfusion, cognitive impairment, mitochondria, mitochondrial dysfunction, Chinese herbal medicine

## Abstract

Chronic cerebral hypoperfusion (CCH) is one of the main pathophysiological markers of cognitive impairment in central nervous system diseases. Mitochondria are cores of energy generation and information process. Mitochondrial dysfunction is the key upstream factors of CCH induced neurovascular pathology. Increasing studies explored the molecular mechanisms of mitochondrial dysfunction and self-repair for effective targets to improve CCH-related cognitive impairment. The clinical efficacy of Chinese herbal medicine in the treatment of CCH induced cognitive impairment is definite. Existed evidences from pharmacological studies have further proved that, Chinese herbal medicine could improve mitochondrial dysfunction and neurovascular pathology after CCH by preventing calcium overload, reducing oxidative stress damage, enhancing antioxidant capacity, inhibiting mitochondria-related apoptosis pathway, promoting mitochondrial biogenesis and preventing excessive activation of mitophagy. Besides, CCH mediated mitochondrial dysfunction is one of the fundamental causes for neurodegeneration pathology aggravation. Chinese herbal medicine also has great potential therapeutic value in combating neurodegenerative diseases by targeting mitochondrial dysfunction.

## 1 Introduction

Cognitive impairment encompasses the entire process from mild cognitive impairment (MCI) to dementia (Dichgans and Leys, 2017). The current global prevalence of MCI among people over 50 years old is more than 15%, and about 43.8 million people are diagnosed with dementia, which is expected to grow to 70 million by 2050 (Gbd Dementia Collaborators, 2019; Bai et al., 2022). With the aggravation of cognitive decline, patients’ ability of daily living is gradually impaired, which brings huge burdens to families and long-term healthcare system (Yang et al., 2013).

Chronic cerebral hypoperfusion (CCH) caused by poor control of vascular risk factors and aging is one of the main pathophysiological markers of cognitive dysfunction in Alzheimer’s disease (AD), vascular cognitive impairment and dementia (VCID), Parkinson’s disease (PD), and other central nervous system diseases (Fitzpatrick et al., 2005; Yu et al., 2022). The underlying neuropathological damage caused by CCH may persist for decades before clinical symptoms of cognitive impairment (Sabayan et al., 2012). Pathological studies showed that hypoxic/hypoperfusion injury signal generated by CCH first affected mitochondria, the “power source” of cells, leading to collapse of cellular energy cycling (Picard and Shirihai, 2022). The persistence of CCH also seriously impaired the self-regulation of mitochondria, which further aggravated neurovascular injury (Coon et al., 2022). Therefore, mitochondrial dysfunction plays a critical part in CCH induced cognitive impairment. Exploring the molecular mechanisms of mitochondrial dysfunction and self-repair after CCH has once again attracted wide attention. From this perspective, more and more studies are looking for effective targets of mitochondrial protection to reduce CCH induced neurovascular injury and improve cognitive impairment.

Traditional Chinese Medicine (TCM) has a long history of treating cognitive impairment. In the first monograph of Chinese materia medica, *Shennong Bencao Jing* (Shennong’s Classic of Materia Medica), a variety of herbs (including *Panax ginseng* C.A.Mey. And *Acorus spurius* Schott) which make people “intelligence” and “unforgettable” were recorded. Many single herb and classical herbal compounds were proved to have good clinical effects on cognitive impairment caused by different causes (Cai and Jiang, 2017; Hou et al., 2022). Animal and cell experiments further confirmed the protective effect on cognition of Chinese herbal medicine by improving CCH status and reducing related neurovascular damage. Among them, the therapeutic mechanisms of many Chinese herbal monomers and compounds are related to mitochondria-mediated apoptotic genes and signal transduction pathways (Zhou et al., 2020). The current study summarized the existed evidences of Chinese herbal medicine targeting mitochondrial dysfunction for improving CCH-related cognitive impairment. Considering the pathological research progress of CCH-related mitochondrial dysfunction, we are going to discuss the potential therapeutic value of Chinese herbal medicine for neurodegenerative diseases, which might provide new directions for future researches.

## 2 Mitochondrial dysfunction: The key point of CCH induced cognitive impairment

### 2.1 Excitotoxicity induced calcium overload: The initiation of mitochondria dysfunction

**FIGURE 1:**
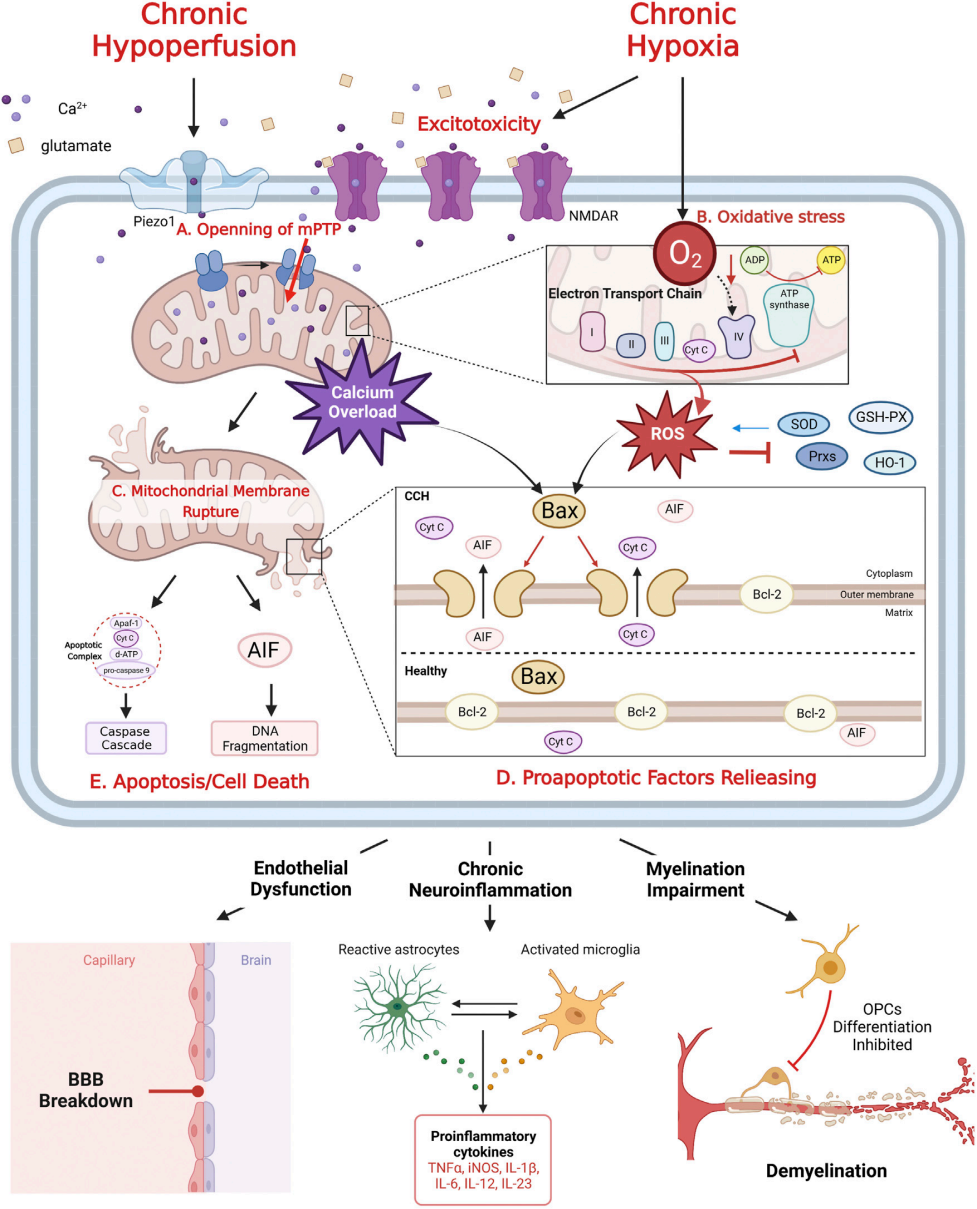
The mechanism of mitochondrial dysfunction after CCH. CCH related excitotoxicity induced mPTP formation **(A)**, which leads to calcium overload. Chronic hypoxia inhibits mitochondrial oxidative phosphorylation, resulting in excessive production of ROS, inhibition of ETC complex and antioxidant enzyme inactivation, eventually oxidative stress **(B)**. Calcium overload and oxidative stress rupture mitochondrial outer membrane **(C)**, trigger the release of pro-apoptotic factors (Cyt C and AIF) **(D)**, which mediate Caspase cascade and DNA fragmentation, respectively, leading to apoptosis/cell death **(E)**. Meanwhile, Bcl-2 family proteins play a key role in regulating the release of anti-apoptotic factors **(D)**. Mitochondrial dysfunction is the key to CCH induced neurovascular injury: endothelial dysfunction, chronic neuroinflammation and myelination impairment.

Excitotoxicity is a form of neuronal death caused by hyperactivity of excitatory amino acids (mainly glutamate) (Velasco et al., 2017). Chronic hypoxia leads to inadequate energy synthesis and significantly reduces adenosine triphosphate (ATP) production in nerve cells by disrupting oxidative phosphorylation. The sodium/potassium pumps on cell membrane are inactivated for the lack of ATP. Following membrane depolarization causes excessive release, re-uptake disorder and finally abnormal accumulation of glutamate. Excess and sustained activation of N-Methyl-D-Aspartate receptors (NMDAR) triggers influx of calcium (Velasco et al., 2017). On the other hand, the hemodynamic abnormality increases intracellular calcium concentration by the opening of mechanically sensitive calcium ions channels (PieZo1) (Coon et al., 2022). Elevated calcium concentration activates the formation of mitochondrial permeability transition pore (mPTP). Once formed, calcium floods into mitochondria causing calcium overload and dissipate mitochondrial membrane potential, which leads to mitochondria swelling and rupture, as well as cellular energy cycle collapse and finally release of apoptotic factors (See Figure 1) (Rottenberg and Hoek, 2017).

Inhibition of continuous activation of NMDAR is efficient to alleviate excitotoxicity induced mitochondrial calcium overload. Specific glutamate antagonists were used to improve ischemia-related neurovascular excitotoxic death, but there is no evidence of their clinical effectiveness (Lai et al., 2014). The non-competitive NMDAR antagonist, memantine, has been widely used in the clinical treatment of cognitive impairment by inhibiting over-activation of NMDAR, while maintaining normal synaptic activity (Matsunaga et al., 2015). Some studies found that memantine also had mitochondrial activation effect independent of anti-excitotoxicity. It can directly enhance the activity of ETC complex and reduce peroxide production under excitotoxic effects (McAllister et al., 2008). On the other hand, memantine also promoted the removal of damaged mitochondria by up-regulating mitophagy flux (Hirano et al., 2019). Edaravone, a clinically used free radical scavenger, could effectively inhibit mitochondrial calcium overload caused by mPTP opening, reduce the production of ROS, promote the expression of antioxidant enzymes, improve mitochondrial energy metabolism disorders and antioxidant capacity, which significantly reduced blood-brain barrier (BBB) damage, and improve cognitive impairment (Takayasu et al., 2007).

### 2.2 Oxidative stress: The key node of mitochondrial dysfunction mediating CCH pathology

Under normal circumstances, reactive oxygen species (ROS) are by-products of mitochondrial aerobic respiration and play important roles in multiple signaling pathways and maintenance of cellular homeostasis (Samoylenko et al., 2013). Normally, excessive ROS can be scavenged by several antioxidizes, such as superoxide dismutase (SOD), peroxiredoxins (Prxs) and glutathione peroxidase (GSH-PX). They can convert ROS into less harmful molecules. Unfortunately, ROS also cause inactivation of antioxidizes under CCH conditions, leading to the persistence of oxidative stress (Powers and Jackson, 2008; Coon et al., 2022). With the process of CCH, the balance between antioxidant enzymes and ROS becomes broken and moves toward oxidative stress (See Figure 1) (Liu and Zhang, 2012).

90% of intracellular ROS under CCH conditions originates from electron transport chain (ETC). Even though transient increased mitochondrial calcium could further elevate energy production (Nakano et al., 2011), excessive mitochondrial calcium uptake following CCH initiated the formation of mPTP (Nichols et al., 2018). Opened mPTP permits entry of not only calcium and other metabolites into the mitochondrial matrix space, which leads to reduction of ATP by uncoupling ETC from ATP synthase activity, cease of oxidative phosphorylation and eventually excessive production of ROS (Pérez and Quintanilla, 2017). Specifically, ATP synthesis on Complex IV is impaired due to insufficient oxygen supply, and the electron transport process is halted on Complex III, leading to over-production of ROS. ROS in turn destroy the activity of Complexes I to IV and collapse mitochondrial respiratory function (Qiao et al., 2002). ROS accumulation further induces free radical mitochondrial damages: over-oxidation of lipids/proteins and deletion of mitochondrial deoxyribonucleic acid (mtDNA) (Zuo et al., 2015).

Mitochondrial oxidative stress disrupts extensive neurovascular coupling, which aggravates hemodynamic disturbances overall cerebral hypoperfusion (Liu and Zhang, 2012). The tight junctions between vascular endothelial cells and the nitric oxide signaling pathway are disrupted by excessive ROS, leading to destruction of the BBB and further decline of vascular self-regulation (Rajeev et al., 2022). In addition, mitochondrial calcium overload triggered by the mechanical damage signal and high levels of ROS impairs the expression of transcription factor Kruppel-like factor 2 (KLF2), which promotes translation of antioxidant, anti-inflammatory factors, and inhibits pro-inflammatory factors releasing. Vast oxidative stress induces chronic neuroinflammation, white matter myelin damage, and ultimately cognitive dysfunction (Jia et al., 2021; Coon et al., 2022).

Mitochondrial-targeted antioxidants might be an effective treatment for cognitive impairment. Butylphthalide, a commonly used clinical mitochondrial protective agent and oxidative stress inhibitor, could significantly reduce the hippocampal mitochondrial ROS level, enhance SOD activity, and improve the learning and memory ability of bilateral common carotid artery occlusion (BCCAO) rats, and also showed good cognitive improvement effects in clinical trials of VCID patients (Chen et al., 2019; Guo, 2019). Edaravone could alleviate the disorder of oligodendrocyte precursor cell differentiation by increasing the activity of SOD and ETC complex (Miyamoto et al., 2013). Alpha-lipoic acid, a natural antioxidant that widely exists in human body, had good permeability of BBB. It showed neuroprotective effect in BCCAO rats by stabilizing mitochondrial membrane potential, reducing excessive production of ROS, and enhancing SOD activity (Zhao et al., 2014).

### 2.3 Mitochondria-related apoptosis/cell death: Essential pathways of neurovascular injury in CCH

Mitochondrial calcium overload and oxidative stress can ultimately rupture mitochondrial outer membrane, which leads to mitochondrial residing proapoptotic factors releasing: cytochrome C (Cyt C) and apoptosis-inducing factor (AIF) (Dave et al., 2011; Galluzzi et al., 2018). Once Cyt C is released into the cytosol, it can assemble with apoptoticprotease-activatingfactor-1 (Apaf-1), pro-caspase-9 and deoxy-ATP to form the apoptotic complex. The complex in turn activates caspase9 and caspase3, initiates the Caspase cascade reaction, and ultimately leads to apoptosis. AIF normally binds with Complex I. Upon its liberation from mitochondria, it exerts DNA fragmentation activities as a part of parthanatos (See Figure 1) (Vakifahmetoglu-Norberg et al., 2017).

Bcl-2 family proteins are major regulators of mitochondrial apoptotic factors releasing: Anti-apoptotic proteins, such as Bcl-2, exist on the mitochondrial outer membrane; pro-apoptotic protein, such as Bax, generally present in the cytoplasm. After receiving the apoptotic signal of calcium influx or ROS, Bax relocates to the mitochondrial outer surface and forms a hole that across both membranes, leading to increase of membrane permeability, thus releasing apoptotic factors (Danial and Korsmeyer, 2004). Besides Bel-2 family proteins, phosphatidylinositol 3-hydroxykinase (PI3K) is also an important cytoplasmic anti-apoptotic cytokine. When it is stimulated by calcium overload and ROS signals, PI3K initiates phosphorylation of protein kinase B (Akt) and Cyclic adenosine phosphate-response element binding protein (CREB) (Uzdensky, 2019). CREB, mostly located in mitochondria, directly promote Bcl-2 expression against Cyt C releasing and inactivate caspase 3, thus preventing the start of mitochondrial apoptosis pathway. On the other hand, activated CREBs also play a neuroprotective role through upregulation of brain derived neurotrophic factor (BDNF) and anti-inflammatory factors in animal model of cerebral ischemia (Zarneshan et al., 2022). ROS can also cause hyperactivation of poly-adenosine diphosphate (ADP)-ribose polymerase 1 (PARP1), an initiator of DNA fragmentation. PARP1 is originally a cytoplasmic DNA repair enzyme, but its activation form mediates cytotoxic effects which inactivates mitochondrial Complex I ∼ III and increases AIF releasing finally leading to DNA fragmentation (Galluzzi et al., 2018).

Mitochondrial dysfunction and structural disruption, an amplified signal of apoptosis/cell death, play a key role in survival and death of nerve cells after CCH (Vakifahmetoglu-Norberg et al., 2017). Inhibiting mitochondrial apoptosis/cell death pathways and reducing the release of mitochondrial proapoptotic factors are essential to alleviate CCH damage and delay cognitive impairment. In oxygen glucose deficiency (OGD) cell models, the natural product 2,4,5-trihydroxybenzaldehyde (TDB) could inhibit the release of pro-apoptotic substances in mitochondria and the activation of the subsequent caspase-9/3 apoptotic pathway by down-regulating miR34a, a negative regulator for Bcl-2 (Liao et al., 2016). Resveratrol is a natural chemical extracted from the rhizome of Polygonum cuspid. (Sun et al., 2014) found that resveratrol could reduce the expression of Bax and Caspase-3, upregulate the expression of Bcl-2, and inhibit mitochondria-related apoptotic pathways in BCCAO rats.

### 2.4 Mitochondrial autoregulation disorder: An aggravating factor of CCH-related cognitive impairment

**FIGURE 2:**
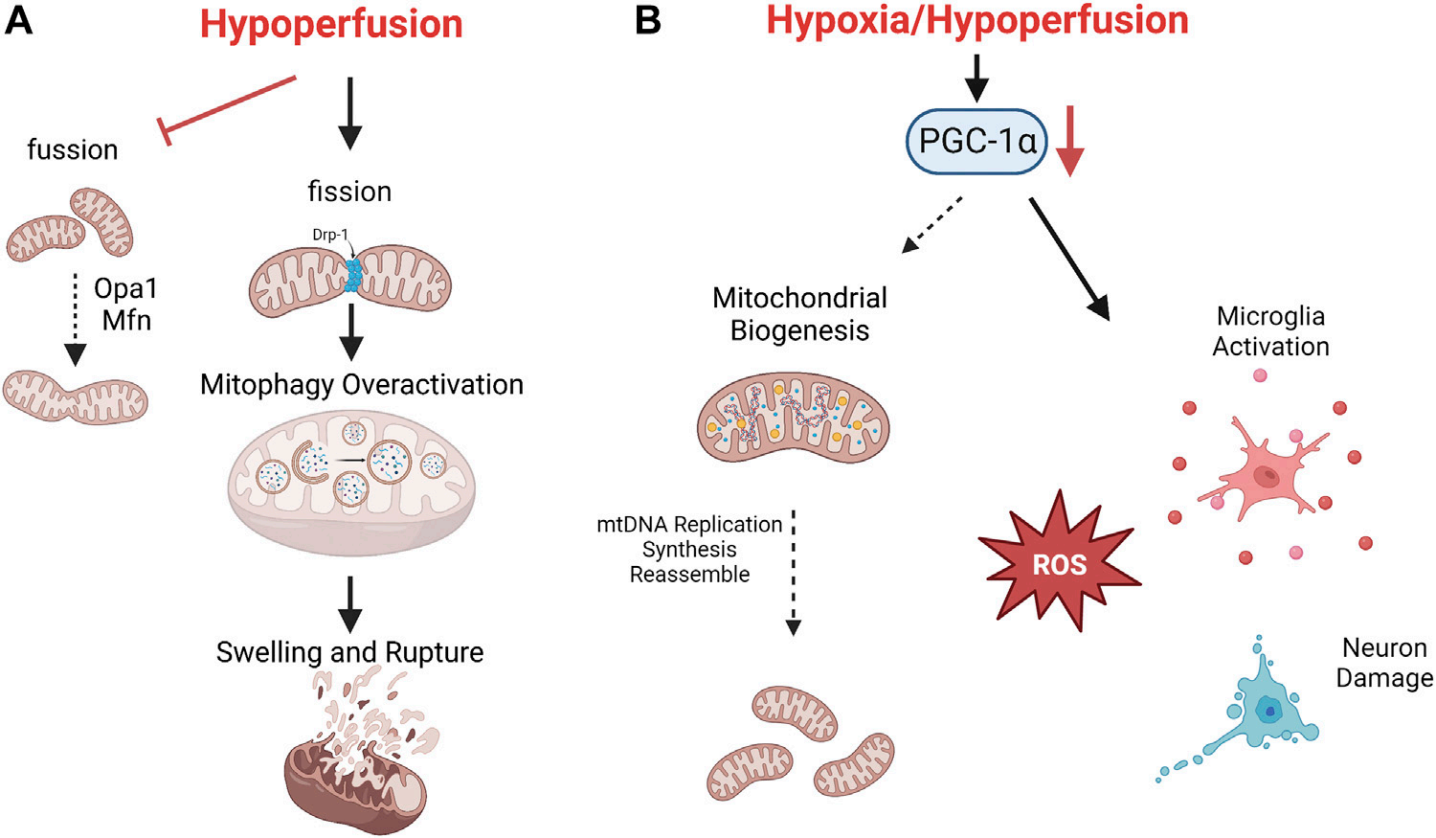
The mechanism of mitochondrial autoregulation disorder after CCH. Consistent hypoperfusion seriously damages mitochondrial dynamics. Mitochondrial fission is aggravated and causes overactivation of mitophagy, which finally leads to swelling and rupture **(A)**. Low level of PGC-1α after CCH promotes oxidative stress, microglia activation and neuron death **(B)**.

Transient hypoxia, calcium influx, and ROS can moderately activate mitochondrial dynamics and mitophagy to maintain partial normal function (Kaur and Sharma, 2022). Mitochondrial dynamics is the dynamic balance of continuous fission and fusion. The dynamic related protein 1 (Drp1) can promote mitochondrial fission, which facilitate mitochondrial autophagy and prevent aggregation of damaged mitochondria. Optic Atrophy protein 1 (Opa1) and mitochondrial fusion proteins (Mfn) promote mitochondrial fusion, inhibit excessive autophagy and prevent further mitochondrial damage (Zacharioudakis and Gavathiotis, 2022). Mitophagy is a selective type of autophagy for removing defective mitochondria. Moderately activated mitophagy can promote mitochondrial self-regulation, improve the antioxidant and anti-inflammatory capacity by increasing KLF2 expression, and alleviate nerve cell damage (Coon et al., 2022). Chronic hypoxia, mitochondrial calcium overload and excessive ROS accumulation may completely inhibit or over-activate mitophagy, leading to imbalance of mitochondrial homeostasis, which further aggravate CCH damage and cognitive impairment (See Figure 2) (Wolf et al., 2019).

Mitochondrial biogenesis refers to a series of molecular processes that trigger mitochondrial replication and subsequent regeneration of fully functional mitochondria. CCH can activate multiple pathways of mitochondrial biogenesis and induce mitochondrial DNA replication, as well as the subsequent synthesis and assemble of lipid/protein molecules for new mitochondria (Cardanho-Ramos and Morais, 2021). The transcription factor peroxisome proliferator-activated receptor (PPAR)-gama coactivator 1-alpha (PGC-1α) is the initiator of mitochondrial biogenesis. PGC-1α recruits additional transcription factors of nuclear encoded genes, which promotes the transcription and translation of various ETC subunits such as ATP synthase, Cyt C, cytochrome oxidase IV, and ROS scavenging enzymes (Kaur and Sharma, 2022). On the other hand, PGC-1α also enhances synaptic plasticity, promotes neuronal survival and repair by up-regulating BDNF, regulating neuronal cholinergic activity and reducing microglia activation. A significant decrease of PGC-1α was observed in CCH animal models, which hindered mitochondrial self-repair, extends the scope of nerve damage, leading to significant impairment of learning and memory ability (Han et al., 2020).

Regulating mitochondrial dynamics, moderately activating mitophagy, and promoting mitochondrial biogenesis are key targets for restoring neurovascular structure and delaying further cognitive decline (Kaur and Sharma, 2022). Rapamycin, mammalian target of rapamycin (mTOR) inhibitor, moderately activated mitophagy as well as inhibited mitochondria-related apoptosis by inhibiting the PI3K/Akt/mTOR pathway in VD rats (Zheng et al., 2021). Nicotinamide adenine dinucleotide (NAD+), a key regulator of cellular oxidative stress signaling pathway, significantly inhibits microglia activation and neuroinflammation after CCH. Recent studies have further demonstrated that NAD+ also played a protective role for mitochondria by activating Sirtuin 1(SIRT 1), one of the targets for upregulating PGC-1α (Zhao et al., 2021). Another free radical scavenger, ferulic acid, could regulate mitochondrial dynamics in ODG cell models by increasing the expression of Drp1 and p-Drp1 and reduce oxidative stress damage (Chen et al., 2017).

## 3 Existed evidences: How can Chinese herbal medicine improve CCH-Related cognitive impairment by targeting mitochondria dysfunction?

### 3.1 Attenuating excitotoxicity for stabilizing mitochondrial membrane potential

The integrity of mitochondrial membrane is fundamental for proper mitochondrial function. As mentioned above, excitotoxicity induced imbalance of ion metabolism (calcium overload) is the initiator in mitochondrial membrane disruption. Antagonism of glutamate excitotoxicity is a potential target for protecting mitochondrial structural integrity and stabilizing mitochondrial membrane potential (Granatiero et al., 2017). (Yang et al., 2022) found that Phlegm-purging Decoction, a classic Chinese herbal compound for dementia (see Table 1), could effectively reduce hippocampal neuron damage by reducing glutamate concentration and NMDAR expression in BCCAO rats. Moreover, Kaixin Powder (see Table 1), another classic compound for amnesia, was proved to improve cognitive impairment in AD and VD animal models. (Li et al., 2021) found that, Kaixin Powder could improve hippocampal mitochondrial functions and alleviate mitochondrial swelling by increasing mitochondrial membrane potential and ATP concentration in multi-infarction dementia rats. Meanwhile, the concentration of glutamate and the phosphorylated NMDAR 1 was also significantly reduced. Further study indicated that, Ginsenoside Rd, the main active ingredient of *Panax ginseng* C.A.Mey., significantly attenuated the glutamate-induced NMDA receptors activation and calcium influx, which effectively stabilizes mitochondrial membrane potential and reduces mitochondrial rupture (Zhang et al., 2008).

**TABLE 1:** Composition of Chinese herbal compounds targeting mitochondria after CCH.

Name of compound translation/Chinese name	Preparation	Composition of herbs scientific name/Chinese name (dosage or ratio)	Animal model/Cell model	References
Phlegm-purging decoction/涤痰汤	Decoction	*Arisaema erubescens* (Wall.)Schott/胆南星(10 g^*^)	Rat model of high-fat diet combined with BCCAO	Yang et al. (2022)
		*Pinellia ternata* (Thunb.) Breit./半夏(10 g)		
		*Citrus reticulata* Blanco/橘红(10 g)		
		*Acorus spurius* Schott/石菖蒲(10 g)		
		*Bambusa tuldoides* Munro/竹茹(10 g)		
		*Citrus aurantium* L./枳实(10 g)		
		*Panax ginseng* C.A.Mey./人参(10 g)		
		*Poria cocos* (Schw.) Wolf/茯苓(15 g)		
		*Zingiber officinale* Roscoe/生姜(10 g)		
		*Glycyrrhiza uralensis* Fisch./甘草(6 g)		
Kai Xin San/开心散	Decoction	*Panax ginseng* C.A. Meyer./人参	Rat model of multi‐infarct dementia 一①Dementia (injecting homologous blood emboli into the right internal carotid artery) ②Neurotoxicity cell model induced by glutamate	Li et al. (2021)
		*Poria cocos* (Schw.) Wol/茯苓		
		*Polygala tenuifolia* Willd./远志		
		*Acorus tatarinoxjuii* Schott/石菖蒲 (Ratio: 3:3:2:2)		
Shenmayizhi decoction/参麻益智汤	Decoction	*Panax ginseng* C.A. Mey/人参	Rat model of high-fat diet combined with BCCAO	Sun et al. (2021)
		*Gastrodia elata* Bl./天麻		
		*Euonymus alatus* Sieb./Thunb./鬼箭羽		
		*Ligusticum chuanxiong* Hort./川芎 (Ratio: 3:3:3:2)		
Zuogui Pill/左归丸	Water-honeyed pill	*Rehmannia glutinosa* (Gaertn.) DC./熟地	Rat model of high-fat diet combined with BCCAO	Zhan et al. (2013), Yu et al. (2014)
		*Dioscorea oppositifolia* L./山药		
		*Lycium barbarum* L./枸杞		
		*Cornus officinalis* Siebold & Zucc./山茱萸		
		*Cyathula officinalis* K.C.Kuan/川牛膝		
		*Cuscuta australis* R.Br./菟丝子		
		Cervi cornus Colla/鹿角胶		
		Testudinis Carapax et Plastrum/龟甲胶 (1 g per pill)		
Qufeng Tongqiao decoction/祛风通窍汤	Decoction	*Ephedra sinica* Stapf/麻黄(10 g)	Rat model of high-fat diet combined with BCCAO	Bai et al. (2014)
		*Pueraria lobata* (Willd.) Ohwi/葛根(30 g)		
		*Whitmania pigra* Whitman/水蛭(5 g)		
		*Pheretima aspergillum* (E.Perrier)/地龙(10 g)		
		*Buthus martensii* Karsch/全蝎(5 g)		
		*Ganoderma lucidum* (Leyss.ex Fr.) Karst./灵芝(10 g)		
		*Acorus tatarinowii* Schott/石菖蒲(20 g)		
Fuzhi capsule/复智胶囊	Capsule	*Polygonum multiflorum* Thunb./何首乌	Rat model of high-fat diet combined with BCCAO	Sun et al. (2017)
		*Rehmannia glutinosa* (Gaertn.) DC./熟地		
		*Cornus officinalis* Siebold & Zucc./山茱萸		
		*Astragalus membranaceus* (Fisch.) Bunge/黄芪		
		*Pueraria lobata* (Willd.) Ohwi/葛根		
		*Ligusticum chuanxiong* Hort./川芎		
		*Prunus persica* (L.) Batsch/桃仁		
		*Acorus tatarinowii* Schott/石菖蒲		
		*Polygala tenuifolia* Willd./远志 (0.5 g per capsule)		
Modified decoction of Rehmanniae/地黄饮子加减方	Chinese medicine particle prescription	*Ziziphus jujuba* var. *Spinosa* (Bunge) Hu ex H.F.Chow/酸枣仁(15 g)	Rat model of high-fat diet combined with BCCAO	Zhou et al. (2020)
		*Polygala tenuifolia* Willd./远志(15 g)		
		*Amomum villosum* Lour./砂仁(15 g)		
		*Dioscorea oppositifolia* L./山药(15 g)		
		*Poria cocos* (Schw.) Wol/茯苓(15 g)		
		*Atractylodes macrocephala* Koidz./白术(15 g)		
		*Eucommia ulmoides* Oliv./杜仲(12 g)		
		*Cyathula officinalis* K.C.Kuan/川牛膝(12 g)		
		*Angelica sinensis* (Oliv.) Diels/当归(12 g)		
		*Myristica fragrans* Houtt./肉豆蔻(12 g)		
		*Drynaria fortunei* (Kunze ex Mett.) J.Sm./骨碎补(12 g)		
		*Rehmannia glutinosa* (Gaertn.) DC./生地黄(12 g)		
		*Paeonia lactiflora* Pall./白芍(12 g)		
		*Panax ginseng* C.A. Mey/人参(10 g)		
		*Epimedium brevicornu* Maxim./淫羊藿(10 g)		
		*Cistanche deserticola* Y.C.Ma/肉苁蓉(10 g)		
		*Cinnamomum cassia* (L.) J.Presl/肉桂(10 g)		
		*Acorus spurius* Schott/石菖蒲(10 g)		
		*Lonicera japonica* Thunb./金银花(9 g)		
		*Cimicifuga heracleifolia* Kom./升麻(8 g)		
		*Schisandra chinensis* (Turcz.) Baill./五味子(8 g)		
		*Arctium lappa* L./牛蒡子(6 g)		
		*Glycyrrhiza uralensis* Fisch./甘草(6 g)		
		*Pueraria lobata* (Willd.) Ohwi/葛根(5 g)		
		*Rosa laevigata* Michx./金樱子(4 g)		
		*Panax notoginseng* (Burkill) F.H.Chen/三七(5 g)		
Naomaitai capsule/脑脉泰胶囊	Capsule	*Panax notoginseng* (Burkill) F.H.Chen/三七	Rat model of high-fat diet combined with BCCAO	Huang et al. (2019)
		Ginkgo biloba L./银杏叶		
		*Angelica sinensis* (Oliv.) Diels/当归		
		Carthamus tinctorius L./红花		
		*Salvia miltiorrhiza* Bunge/丹参		
		*Crataegus pinnatifida* Bunge/山楂		
		*Spatholobus suberectus* Dunn/鸡血藤		
		*Panax ginseng* C.A.Mey./红参		
		*Chrysanthemum morifolium* Ramat./菊花		
		*Haliotis diversicolor* Reeve/石决明		
		*Polygonum multiflorum* Thunb./何首乌		
		*Acorus spurius* Schott/石菖蒲		
		*Pueraria lobata* (Willd.) Ohwi/葛根		
		(0.5 g per capsule)		
Shenzhi Jiannao formula/参知健脑方	Decoction	Panax ginseng C.A. Mey/人参	Hypoxia model of aged rats	Wu et al. (2017)
		*Paeonia lactiflora* Pall./赤芍		
		Anemarrhena asphodeloides Bunge/知母 (Ratio: 2:2:1)		

*g gram.

### 3.2 Lessening mitochondrial oxidative stress impairment

**FIGURE 3:**
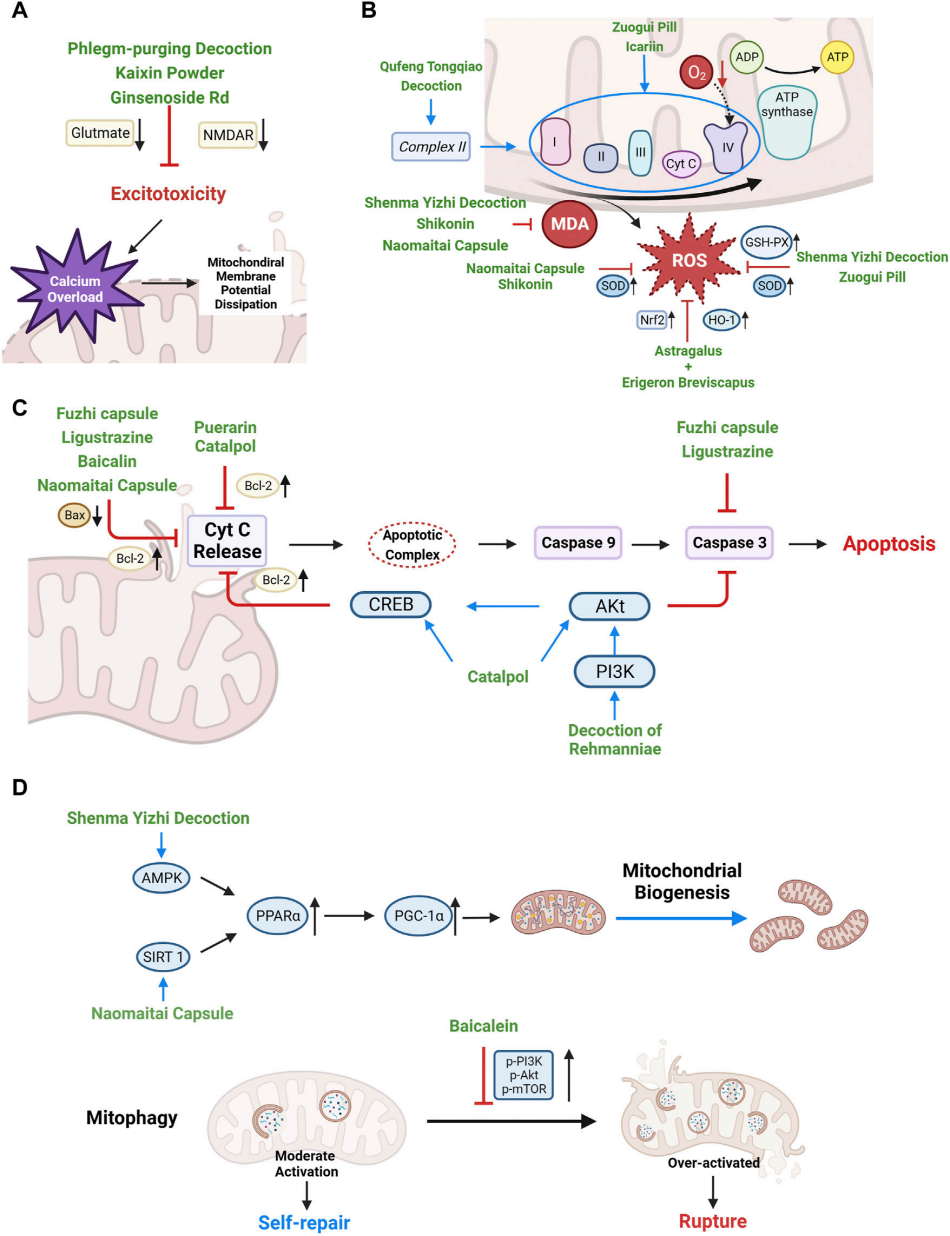
Therapeutic mechanisms of Chinese herbal medicine targeting mitochondrial dysfunction and self-regulation disorder after CCH. **(A)** Stabilizing mitochondrial membrane potential, reducing mitochondrial calcium overload, and preventing mitochondrial rupture by alleviating excitotoxicity. **(B)** Enhancing antioxidant enzyme activity, protecting ETC to reducing ROS production. **(C)** Regulating the expression of Bcl-2 and Bax to reduce the release of mitochondrial pro-apoptotic factors. **(D)** Upregulating PGC-1α and inhibiting mitophagy overactivation to restore mitochondrial self-regulation.

Under CCH conditions, 90% of intracellular ROS is derived from mitochondria, which are also the key sites of oxidative stress damage (Nissanka and Moraes, 2018). Enhancing the antioxidant defense capacity of mitochondria and improving the activity of ETC complex are important means to reduce ROS production and alleviate oxidative stress. Current studies shows that a variety of Chinese herbal medicine exert anti-oxidative stress effects under CCH condition. (See Figure 3B)

#### 3.2.1 Enhancing antioxidant capacity of mitochondria

Shikonin is a naphthoquinone compound extracted from the root of *Arnebia euchroma* (Royle) Johnst. Recent studies showed that Shikonin was a promising antioxidant. (Li et al., 2018) found that Shikonin significantly improved spatial learning and memory in BCCAO rat models. Compared with the model group, Shikonin enhanced the activity of SOD, reduced the content of lipid peroxides malondialdehyde (MDA) and improved the mitochondrial membrane potential in hippocampus. (Sun et al., 2021) indicated that Shenma Yizhi Decoction (see Table 1) could reduce the level of MDA and increased the activity of GSH-PX and SOD in BCCAO rats. In OGD model of PC12 cells, the endogenous antioxidant stress regulator nuclear factor E2 related factor 2 (Nrf2) in mitochondria was activated and mediated the transcriptional activation of antioxidant enzyme genes, such as heme oxygenase-1 (HO-1) (Waz et al., 2021). (Yan et al., 2022) found Astragalus and Erigeron Breviscapus combination (Astragaloside: chlorogenic acid: erigeron breviscapine = 10 μmol/L: 75 μmol/L: 40 μmol/L) could decrease ROS level of OGD-PC12 cells by increasing the expression of Nrf2 and HO-1. In the meantime, the combination can also reduce mPTP opening and stabilize mitochondrial membrane potential. Naomaitai capsule (see Table 1), a Chinese compound for cerebral ischemia, improved cognitive function of BCCAO rats. Further studies found that Naomaitai capsule improved periventricular white matter damage caused by long-term hypoxia by reducing inflammatory factors and MOD content, increasing SOD expression (Huang et al., 2019).

ADP/oxygen (O) ratio and oxidative phosphorylation rate (OPR) were used to assess the overall antioxidant function of mitochondria. Reduce of ADP/O and OPR represents impaired mitochondrial oxidative phosphorylation, reduced energy production and dysfunctional energy transfer (Navet et al., 2006). Baicalein is a natural flavonoid from *Scutellaria baicalensis* Georgi and an excellent free radical scavenger (Okatani et al., 2003). (He et al., 2009) found that baicalein could restore the coupling of ETC, increase ATP synthesis and improve the antioxidant capacity of mitochondria by increasing the ADP/O ratio and OPR in mitochondria of BCCAO rats. Zuogui Pill (see Table 1), an ancient Chinese herbal compound, showed cognitive protection effect in BCCAO rats (Li et al., 2010). (Zhan et al., 2013) further indicated that Zuogui Pill alleviated CCH related cognitive impairment by improving ability of mitochondrial free radical scavenging (increasing ADP/O ratio) and antioxidant enzymes (GSH-PX and SOD).

#### 3.2.2 Protecting ETC complex

(Yu et al., 2014) found that Zuogui Pill could effectively reduce ROS production and improve mitochondrial function by restoring the activity of Complex IV in BCCAO rats. (Bai et al., 2014) indicated that Qufeng Tongqiao Decoction (see Table 1) could promote the expression of *Compex II* gene, which significantly enhanced the overall activity of ETC complex and improve mitochondrial dysfunction. In the cell model of oxygen free radical damage caused by Fe^2+^/vitamin C, (Li et al., 2005) found that Preconditioning icariin (extracted from *Epimedium brevicornu* Maxim) effectively prevented the damage of Complex II-IV and accumulation of MDA.

### 3.3 Inhibiting mitochondria-related apoptosis/cell death pathways

Regulating Bel-2 family proteins is an effective target to block mitochondria-related apoptosis/cell death, which plays an important role in alleviating ischemic nerve cell injury (Liao et al., 2016). Puerarin is an isoflavone compound extracted from *Pueraria lobata* (Willd.) Ohwi. (Gegen). (Chang et al., 2009) found that puerarin can improve the learning and memory ability of BCCAO rats by promoting the expression of Bcl-2 gene and reducing apoptosis. (Sun et al., 2017) found that Fuzhi capsule (see Table 1), a Chinese patent preparation with the effect of “invigorating kidney essence”, effectively reduced apoptosis of hippocampal neuron cells in BCCAO rat models by increasing Bcl-2 while inhibiting the expression of Bax and Caspase-3. (Zhao et al., 2018; Li and Shu, 2020) also indicated that ligustrazine (extracted from *Ligusticum chuanxiong* Hort.) inhibited the mitochondrial apoptotic pathway by reducing the Bax/Bcl-2 ratio and Caspase-3 in OGD-PC12 cells. The study of (He et al., 2009) showed that Baicalin (extracted from *Scutellaria baicalensis* Georgi) can not only reduce the Bax/Bcl-2 ratio of BCCAO model rats, but also the release of mitochondrial Cyt C. (Zhou et al., 2020) found that Decoction of Rehmanniae (see Table 1), another Chinese herbal compound, could enhance the anti-apoptosis mediated by PI3K/Akt/CREB pathway and reduce the activation of caspase 3, thus prevent mitochondrial related apoptosis. (Zhang et al., 2017) proved that in BCCAO models, catalpol, the main active component of *Rehmannia glutinosa* (Gaertn.) DC., increased the level of Bcl-2, p-Akt and p-CREB in oligodendrocytes, which directly reduced white matter damage. In animal models of CCH, Naomaitai capsule increased intracellular Bcl-2 expression while decreased Caspase-3 and Bax (Huang et al., 2019) (see Figure 3C).

### 3.4 Restoring mitochondrial self-regulation

Chinese herbal medicine has shown promising potential in activating PGC-1α upstream pathway to promote mitochondrial biogenesis (Kaur and Sharma, 2022) (see Figure 3D). Adenosine monophosphate (AMP)-activated protein kinase (AMPK) is a cellular energy sensor and the guardian of mitochondria. After receiving the oxidative stress signal, AMPK upregulates the expression of PGC-1α, promotes the increase of mitochondrial antioxidants and initiates mitochondrial biogenesis (Calegari et al., 2011). (Sun et al., 2021) found that Shenma Yizhi Decoction could significantly increase the mRNA and protein levels of AMPK, PPARα, PGC-1α, in BCCAO rats, eventually increased the level of mitochondrial synthesis protease (ATP-5A), repaired mitochondrial structure. Another upstream stimulator of PGC-1α is SIRT 1, which is responsible for sensing ROS and inducing mitochondrial biogenesis, through activation of PPARs and PGC-1α. (Chang et al., 2014) indicated that puerarin (*Pueraria lobata* (Willd.) Ohwi) can activate SIRT 3/PGC-1α and reduce the number of apoptotic cells in BCCAO rats.

Inhibiting the overactivation of mitophagy is also essential for promoting mitochondrial self-repair (Lan et al., 2018). In animal models of ischemia-reperfusion, (Yang et al., 2019) showed that Baicalin can reduce the hyperactivation of mitophagy in subacute ischemia-reperfusion injury by promoting PI3K/Akt/mTOR pathway in order to increase the ratio of Bcl-2/Bax, reduce cell apoptosis, and improve neurological deficits.

## 4 Potential direction: Chinese herbal medicine targeting mitochondrial dysfunction for CCH related neurodegeneration pathology

CCH is an independent exacerbating reason for impairment in learning and memory of PD and AD (Eisenmenger et al., 2022; Fan et al., 2022). CCH mediated mitochondrial dysfunction is one of the fundamental causes for both neurovascular damages, as well as neurodegeneration pathology aggravation. Mitochondrial repairment is a future therapeutic target for alleviating neurodegenerative disorder (Kessas et al., 2022).

Recent study (He et al., 2019) found that hippocampal mtDNA deletion in the CCH model were accompanied by increased α-synuclein level. Physiologically, α-synuclein involves regulation mitochondrial membrane permeability, energy metabolism, and mitochondrial dynamics. In addition, excessive accumulation of α-synuclein is not just the signature of PD, but can also lead to increase of amyloid-β (Aβ) and tau protein aggregation, which is the core of AD pathology (Fathy et al., 2022). Excessive accumulation of α-synuclein, Aβ and tau protein can further aggravate mitochondrial dysfunction and induce apoptosis/cell death (Zaretsky and Zaretskaia, 2020; Nam et al., 2022). (Wu et al., 2017) observed that accumulation of brain amyloid precursor protein (APP) and Aβ1-42, inactivation of mitochondrial cytochrome oxidase, as well as increase of mtDNA oxidative damage product 8-hydroxydeoxyguanosine (8-OHdG) in the anoxic model of aged rats. Meanwhile, Shenzhi Jiannao Formula (see Table 1) could reduce the level of APP, Aβ1-42 and 8-OHdG, so as to protect mitochondrial function and improve learning and memory ability of rats. Exploring the effect of Chinese herbal medicine on mtDNA deletion caused by chronic ischemia/hypoxia may be a potential direction to further exploration of neurodegeneration related cognitive impairment.

Mitochondrial oxidative stress also showed close correlation to production and aggregation of disordered proteins (including α-synuclein, Aβ and tau protein). High level of mitochondrial-generated ROS created deleterious modifications environment for normal proteins, lipids, RNA and DNA which exacerbated AD and PD pathology (Korovesis et al., 2023). In AD models, CCH is the precondition of Aβ aggregation. Combination of calcium and ROS production under Aβ stimulation induces the opening of mPTP and mitochondrial dysfunction (Shevtsova et al., 2017). Blocker of mPTP opening (such as inducing cyclophilin D deficiency) could improve mitochondrial function and learning/memory in aged AD mouse models (Du et al., 2008). In PD model, mtDNA deletion in the substantia nigra of PD patients were caused by oxidative, which was induced by mitochondrial Complex mutation and superoxide overproduction. Antioxidants showed promising effect in reducing loss of dopaminergic neurons by increasing mtDNA copy numbers (Angelova and Abramov, 2018). As described earlier, many Chinese herbal active compounds, such as shikonin, astragaloside, chlorogenic acid, erigeron breviscapine, baicalein and icariin, showed good potential in lessening oxidative stress in CCH models. Their antioxidative effect for repairing mitochondrial function under AD and PD pathology worth further exploration.

On the other hand, mitochondrial depletion due to calcium dysregulation and hyperactive mitophagy following glutamate excitotoxicity induced dendritic atrophy in AD and PD models, which is an early sign of neurodegeneration (Verma et al., 2022). Mitochondrial calcium uniporter (MCU) conditional knockout and application of MCU inhibitors both showed potential effect of normalizing mitochondrial calcium flux against dendritic atrophy in neurodegenerative models and restoring neuron viability in OGD models (Nichols et al., 2018; Verma et al., 2022). As mentioned above, Ginsenoside Rd had good effect in alleviating calcium influx for stabilizing mitochondrial membrane potential. Further studies could investigate its calcium regulation effect in AD or PD models.

## 5 Conclusion and perspectives

Mitochondria are the energy center and information processor of cells. Their dysfunction plays a key role in CCH-mediated neurovascular pathology and cognitive impairment. Protecting the integrity of mitochondrial structure/function and restoring mitochondrial self-regulation are effective targets for alleviating CCH-induced cognitive impairment. Modern clinical research and experiments have confirmed the precise role of Chinese herbal medicine in improving CCH status and cognitive impairment. In recent years, modern TCM researchers have further clarified the therapeutic mechanism of Chinese herbal medicine targeting mitochondrial dysfunction to improve CCH-induced cognitive impairment in animal and cellular models using modern technology. Current evidence showed that Chinese herbal compounds and monomers can protect the structural and functional integrity of mitochondria for energy metabolism reconstruction by reducing calcium overload after excitotoxicity, enhancing ETC complex activity, restoring mitochondrial self-regulation. Monomers of Chinese herbs showed good effect in antioxidant and anti-excitotoxicity (discussed in section 4). Puerarin, ligustrazine, Baicalin and catalpol showed clear anti-apoptosis effect. Among them, puerarin and baicalin also promoted mitochondrial self-repair by regulating mitochondrial dynamics and mitophagy.

Significantly, Chinese herbal compounds produced under the guidance of TCM “syndrome differentiation and treatment” theory showed clear efficacy in improving mitochondrial dysfunction and treating cognitive impairment after CCH, which provide new ideas for mitochondrial targeted therapy for neurodegenerative diseases. As mentioned above, (Wu et al., 2017; Li et al., 2021; Sun et al., 2021)conducted studies on therapeutic mechanisms of Kaixin Powder, Shenma Yizhi Decoction, and Shenzhi Jiannao Decoction targeting mitochondrial dysfunction. They used animal models to explore the optimal dosage and proportion for cognitive improvement and reported a standardized preparation process to reduce the differences in efficacy caused by the quality of herbs and processing techniques. The results indicated that these dose-optimized compounds had multi-target and multi-pathway regulatory effects on mitochondrial dysfunction after CCH. In order to further clarify the influence of different herbal combinations and reduce the non-pharmacodynamic factors, it is necessary to conduct pharmacodynamic studies to explore the best herbal compatibility and dose for a specific mitochondrial indicator. (Waz et al., 2021) screened three monomers with cooperative effect on stabilizing mitochondrial membrane potential from the combination of Astragalus injection and Brezhanhua injection. Therefore, they create a new compatibility composed of these three monomers and determined the best concentration for each element. Chinese herbal active component compatibility can not only retain the overall therapeutic advantages of compounds, but also overcome the shortcomings of traditional herbal preparations: Complex chemical composition and unstable effect. Therefore, further researches on the compatibility and optimal ratio for active components from Chinese herbal medicine is the basic method to clarify the relationship between disease/symptom, compound and curative effect index, and also important ways to realize the optimization of TCM compound preparations.

## Glossary

AMP: adenosine monophosphate
ATP: adenosine triphosphate
AMPK: AMP-activated protein kinase
Aβ: amyloid-β
APP: amyloid precursor protein
AD: Alzheimer’s disease
BCCAO: bilateral common carotid artery occlusion
BDNF: brain derived neurotrophic factor
BBB: (blood-brain barrier)
CCH: chronic cerebral hypoperfusion
CREB: cyclic adenosine phosphate-response element binding protein
Cyt C: cytochrome c
Drp1: dynamic related protein 1
ETC: electron transport chain
GSH-PX: glutathione peroxidase
KLF2: Kruppel-like factor 2
MDA: malondialdehyde
MCI: mild cognitive impairment
MCU: mitochondrial calcium uniporter
mtDNA: mitochondrial deoxyribonucleic acid
Mfn: mitochondrial fusion proteins
mTOR: mammalian target of rapamycin
mPTP: mitochondrial permeability transition pore
NMDAR: N-Methyl-D-Aspartate receptors
HO-1: heme oxygenase-1
NAD+: nicotinamide adenine dinucleotide
Nrf2: nuclear factor E2 related factor 2
Opa1: optic atrophy protein 1
OGD: oxygen glucose deficiency
PD: Parkinson’s disease
Prxs: peroxiredoxins
PI3K: phosphatidylinositol 3-hydroxykinase
Akt: protein kinase B
ROS: reactive oxygen species
SOD: superoxide dismutase
TCM: Traditional Chinese Medicine
PPAR: transcription factor peroxisome proliferator-activated receptor
PGC-1α: PPAR-gama coactivator 1-alpha
VCID: vascular cognitive impairment and dementia
TDB: 2,4,5-trihydroxybenzaldehyde
8-OHdG: 8-hydroxydeoxyguanosine
